# Cell behaviour on a polyaniline nanoprotrusion structure surface

**DOI:** 10.1186/1556-276X-9-566

**Published:** 2014-10-11

**Authors:** Hyoryung Nam, Taechang An, Geunbae Lim

**Affiliations:** 1 Department of Integrative Biosciences and Biotechnology, Pohang University of Science and Technology, Pohang, Korea; 2 Department of Mechanical Design Engineering, Andong National University, Andong, Korea; 3 Department of Mechanical Engineering, Pohang University of Science and Technology, Pohang, Korea

**Keywords:** Polyaniline, Nanoprotrusion structure, Cell behaviour, Osteoblast

## Abstract

The extracellular matrix provides mechanical support and affects cell behaviour. Nanoscale structures have been shown to have functions similar to the extracellular matrix. In this study, we fabricated nanoprotrusion structures with polyaniline as cell culture plates using a simple method and determined the effects of these nanoprotrusion structures on cells.

## Background

Most of the cells in the human body require adhesion to surfaces for survival. Therefore, cells generate extracellular matrix (ECM), which facilitates their adherence to a surface and influences their behaviour, such as orientation, proliferation, migration and differentiation [1,2]. In addition, the nanoscale topography of the surface enhances cellular protein adsorption and affects cell behaviour, similar to the ECM [3].

Several studies regarding the fabrication of nanostructures that may influence cell behaviour have been conducted. Teixeira et al. [4] fabricated nanogrooves on silicon using electron-beam lithography. This lithography process allows the generation of diverse nanoscale patterns, such as further groove patterns, protrusions, holes and shapes [5,6]. However, these fabrication processes are complex, expensive and time-consuming. The materials used are also restricted: a silicon wafer is normally used. It is also largely non-transparent, difficult to handle, and is unsuitable for use in routine cell culture in the laboratory.

To address the reproduction and non-transparency problems, Li et al. [7] used polydimethylsiloxane (PDMS), which is a transparent and easy-to-handle material for moulding. Although the moulding accuracy is high, additional processes are required to make a nanoscale mould.

Here, we investigated a readily fabricated polyaniline nanostructure as a cell culture surface. Polyaniline, consisting of aniline polymerised using simple methods, is an interesting and commonly used material because it is an organic polymer that can conduct electricity. Furthermore, polyaniline has a number of advantages, including simple polymerisation, low cost, good environmental stability, controllable electrical conductivity and the ability to polymerise under various conditions [8]. It can be synthesised on many surfaces and applied in various fields, including electrodes, sensors and other biomedical applications [9]. Here, we evaluated the behaviours of MC3T3-E1 cell on a structured polyaniline surface.

## Methods

### Preparation of polyaniline nanoprotrusion structured surface

Polyaniline nanostructures were synthesised on commercial 12-well tissue culture plates (TCP; BD Falcon, San Jose, CA, USA) using the dilute polymerisation method [10]. First, 1 M HClO_4_ (Samchun Chemical, Seoul, Korea), 0.0067 M ammonium persulphate (Sigma-Aldrich, St. Louis, MO, USA), 0.01 M aniline (Sigma-Aldrich, St. Louis, MO, USA) and deionised (DI) water were mixed with an orbital shaker at 4°C for 12 h. The samples were then washed in DI water to remove any residue.

To assess the topographic effects (i.e. with or without nanostructures), the prepared polyaniline nanoprotrusion structure surface was rubbed at saturation level with a rubberised cloth to flatten all protrusions [11].

### Surface properties of polyaniline nanoprotrusion structure surface

The samples were coated with platinum (Pt) and the surface topography was observed by scanning electron microscopy (SEM; Hitachi, Tokyo, Japan). The surface roughness was also measured by atomic force microscopy (AFM; Seiko Instruments Inc., Chiba, Japan).

Surface properties were evaluated by measuring the contact angle of the samples, captured with a camera (Nikon, Tokyo, Japan). The DI water droplet volume was 5 μL. Measurements were performed in five different areas of each sample and analysed using the ImageJ software (National Institutes of Health, Bethesda, MD, USA).

### Cell seeding and culture

The samples were sterilised under UV light for 1 day. MC3T3-E1 osteoblasts were seeded at 0.5 × 10^4^ cells/well and cultured for 7 days in α-MEM supplemented with 10% foetal bovine serum (FBS) and 1% penicillin/streptomycin (P/S) at 37°C, 5% CO_2_. The medium was replaced every 3 days.

### Cell cytotoxicity and morphology

Cell cytotoxicity was verified by live/dead staining. Live cells were stained with calcein-AM (Anaspec, San Jose, CA, USA) and dead cells with ethidium homodimer-1 (EthD-1; Anaspec) after 1 and 7 days in culture. Images were obtained by fluorescence microscopy. The live cells were stained green and dead cells were stained red.

The cell cytoskeleton was stained to examine cell morphology. The actin filaments were stained with phalloidin (Sigma-Aldrich, St. Louis, MO, USA), the nucleus was stained with 4′,6-diamidino-2-phenylindole (DAPI; Golden Bridge, City of Industry, CA, USA). The actin filaments are shown as green and the nucleus as blue on fluorescence micrographs. ImageJ was used to measure the average adhesion area (μm^2^) of each cell.

For more detailed observation of cell morphology, surface-attached cells were visualised by SEM. The samples were fixed in 4% formaldehyde. Following ethanol dehydration and drying [12], the samples were coated with Pt.

### Cell proliferation and differentiation

We measured total DNA levels in MC3T3-E1 cells to determine their proliferation. TRIzol reagent (Invitrogen, Carlsbad, CA, USA) was added to each sample and DNA was extracted according to the manufacturer’s instructions. DNA was quantified using a NanoDrop 2000 (Thermo Scientific, Waltham, MA, USA). The absorbances at 260 and 280 nm of appropriately diluted samples were measured on days 1, 3, 5 and 7 after cell seeding.

Osteoblasts were cultured in medium containing l-ascorbic acid (50 μg/mL) and β-glycerophosphate (10 mM) for 7 days. Osteogenesis-related gene expression was quantified by quantitative real-time reverse transcriptase polymerase chain reaction (qRT-PCR). Total RNA was extracted from samples using TRIzol reagent, converted into complementary DNA (cDNA) using SuperScript II reverse transcriptase (Invitrogen, Carlsbad, CA, USA) and analysed using a LightCycler 2.0 instrument (Roche, Indianapolis, IN, USA). The messenger RNA (mRNA) levels of type I collagen (COL-I), alkaline phosphatase (ALP) and runt-related transcription factor 2 (Runx2) were normalised relative to that of GAPDH as a control.

## Results and discussion

### Surface properties of polyaniline nanoprotrusion structure surface

**Figure 1:**
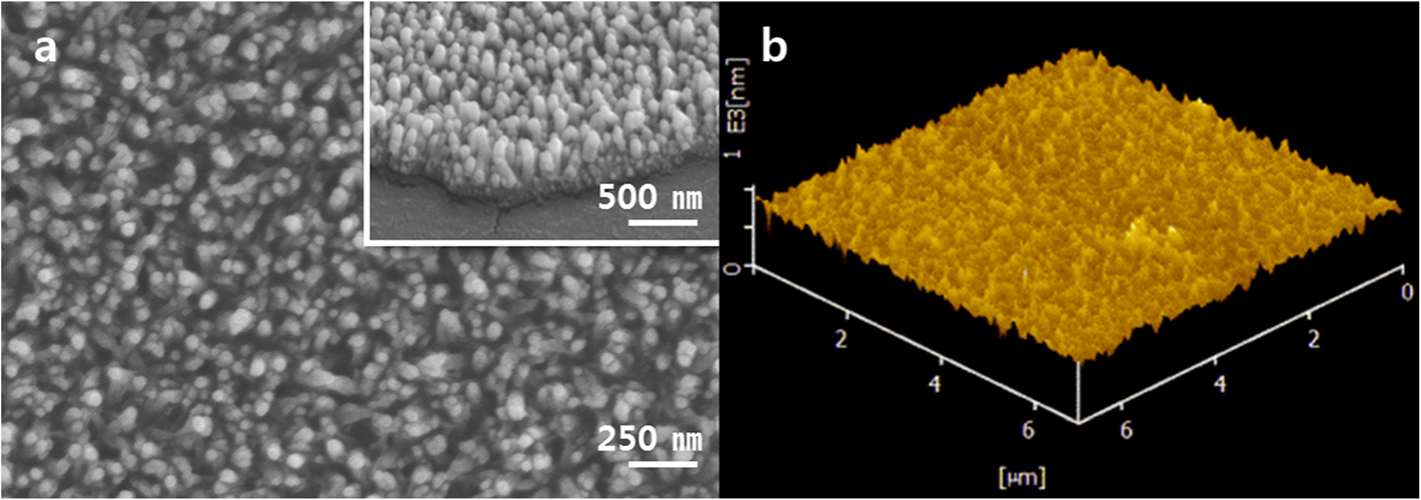
**Surface topography images and 3D image of a PANI nanostructure-coated TCP plate. (a)** SEM front view, the scale bar represents 250 nm; the inset image is a side view in which the scale bar indicates 500 nm. **(b)** AFM 3D image of surface roughness; the scanned area was 6 × 6 μm.

**Figure 2:**
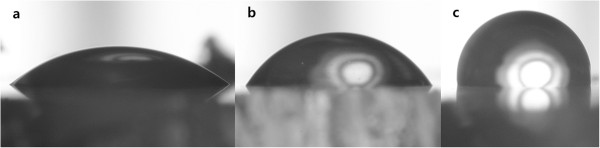
**Photographs of water droplets on the surfaces. (a)** Polyaniline nanoprotrusion structure surface. **(b)** Tissue culture plate. **(c)** Rubbed polyaniline nanoprotrusion structure surface.

We measured the polyaniline nanoprotrusion structure surface (PNS) properties. Figure 1 shows a uniform coating of a polyaniline nanoprotrusion structure on the surface. Figure 1a shows the front; the inset shows a side view (tilted at 30°). The topography after polymerisation shows aligned nanoprotrusions with an average diameter of 44.18 ± 14.62 nm and height of 140.64 ± 46.40 nm. Figure 1b is an AFM 3D image of the PNS and shows a surface roughness (Ra) of 4.154E + 01 nm.Figure 2 shows the contact angle between the water and the PNS. The TCP and the rubbed polyaniline nanoprotrusion structure surface (RPNS) are controls for cell culture and the effects of nanostructure, respectively. The contact angle of the PNS was 37.79° ± 5.33° and that of the TCP was 66.41° ± 5.30°. Thus, the contact angle of the PNS was ~30° less than that of the TCP. Dilute polymerisation produced a more hydrophilic surface than commercial TCP. The RPNS had no nanoprotrusions; its value was 97.82° ± 2.11°. The contact angles of the surfaces with and without nanoprotrusions differed. The RPNS was more hydrophobic than were the PNS and TCP.

### Cell behaviour

**Figure 3:**
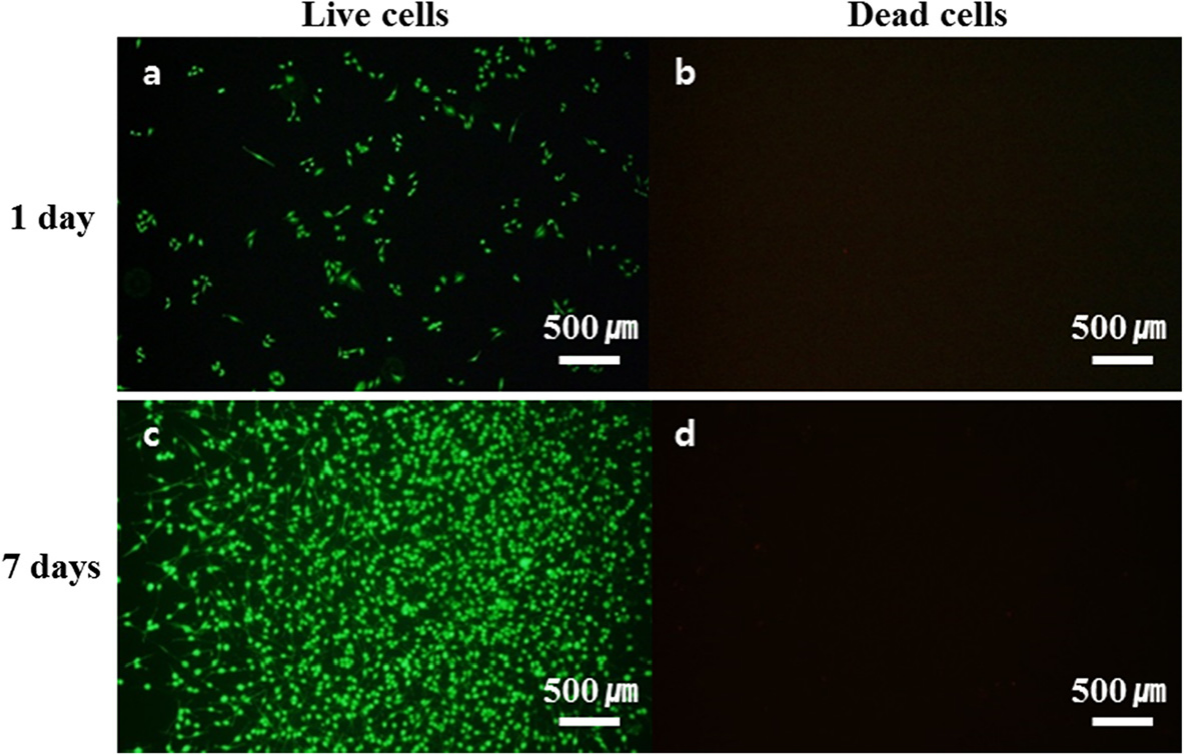
**Fluorescence-stained live/dead MC3T3-E1 cells.** Live cells in **(a)** and **(c)** are indicated by green fluorescence staining with calcein-AM. Dead cells in **(b)** and **(d)** are indicated by red staining with EthD-1. **(a)** and **(b)** are 1 day after seeding, and **(c)** and **(d)** are 7 days after seeding. Scale bars = 500 μm.

**Figure 4:**
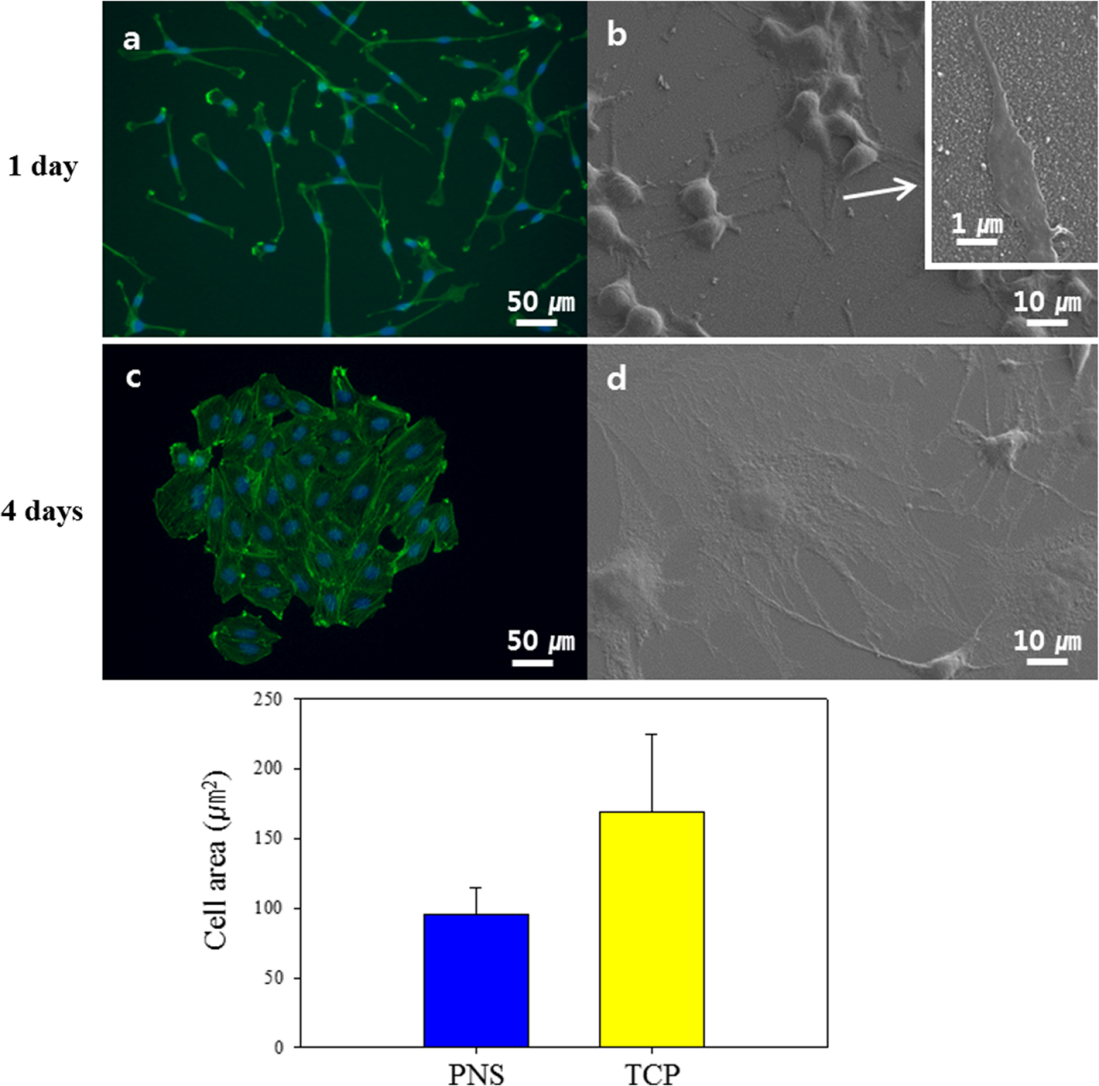
**Fluorescence images of the morphology of MC3T3-E1 cells adhering to the surfaces 4 days after seeding. (a** and **b)** Polyaniline nanoprotrusion structure surface (PNS). **(c** and **d)** Tissue culture plate (TCP). **(a)** and **(c)** show DAPI and phalloidin staining. **(b)** and **(d)** show SEM images of the morphology of cell adhesion to the surfaces. The inset in **(b)** is a higher-magnification image of filopodia on PNS. The bottom graph shows the area of osteoblast cell adhesion. The scale bars in **(a)** and **(c)** are 50 μm, those in **(b)** and **(d)** are 10 μm, and the scale bar in the inset image in **(b)** is 1 μm.

Polyaniline was polymerised on the TCP and used as cell culture plates immediately after sterilisation. Following polyaniline polymerisation, plates were examined for any remnant aniline monomer or solvents that could affect cell viability (Figure 3). Polyaniline shows good biocompatibility but aniline, the monomer, is cytotoxic. Fluorescence images illustrated the condition of osteoblasts on the PNS. Only live cells were present 1 day after cell seeding, and no remnant cytotoxic material was detected (Figure 3a,b). No cytotoxicity was observed after 7 days under typical cell culture conditions (Figure 3c,d). There was no cytotoxicity from polymerisation residue. In addition, the polyaniline was apparently structurally and chemically stable under normal cell culture conditions.As shown in Figure 4, osteoblasts grown on the PNS had a distinct morphology in comparison with cells grown on the TCP. The cells attached to the TCP were round in shape (Figure 4c). However, on the PNS, the cells had a unique adhesion morphology. The long axis of the cells was aligned with the coating. Cells on the PNS showed a smaller area of adhesion to the surface. The conditions of adhesion were markedly different between the TCP and the PNS. There appeared to be no connection between the cells on the PNS based on phalloidin staining (Figure 4a). However, SEM images showed that the cells were closely interconnected. Cells on the TCP were stable, and the rounded, spread cells were connected to adjacent cells. The average adhesion area of cells was calculated using ImageJ, and a greater than twofold difference was observed between the TCP and the PNS (Figure 4, bottom graph).

**Figure 5:**
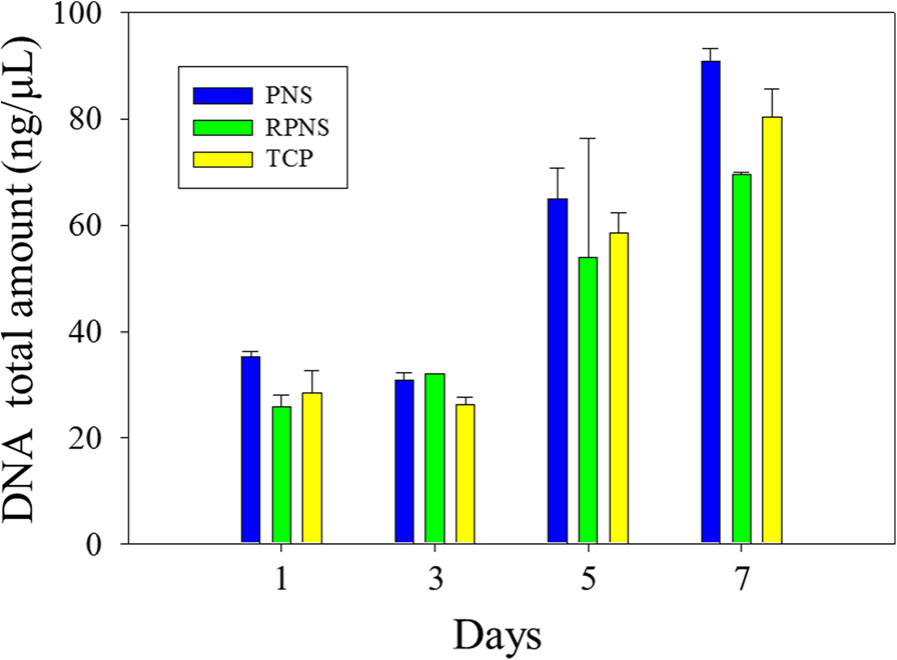
**Total DNA quantities in MC3T3-E1 cells at 1, 3, 5 and 7 days.** The blue bar is the polyaniline nanoprotrusion structure surface (PNS); the green bar is the rubbed polyaniline nanoprotrusion structure surface (RPNS); and the yellow bar is the tissue culture plate (TCP).

Figure 5 shows cell proliferation. The total DNA levels were used to assay cell adhesion on day 1, and cell proliferation during the 7-day culture period. DNA quantification indicated that osteoblasts on the PNS exhibited increased adhesion and proliferation compared to the other samples. This may explain the difference in contact angle. Wei et al. investigated osteoblast adhesion according to water contact angle - osteoblast adhesion increased with increasing surface hydrophilicity within a contact angle range of 0° to 106°. Cells tend to show greater adhesion and proliferation on more hydrophilic surfaces [13]. Denis et al. reported similar results, in which proliferation increased with increasing surface hydrophilicity [14]. The PNS was more hydrophilic than the TCP and RPNS (Figure 2). The contact angle on the PNS is affected by the surface topography, which is itself a result of the presence of nanoprotrusions.

**Figure 6:**
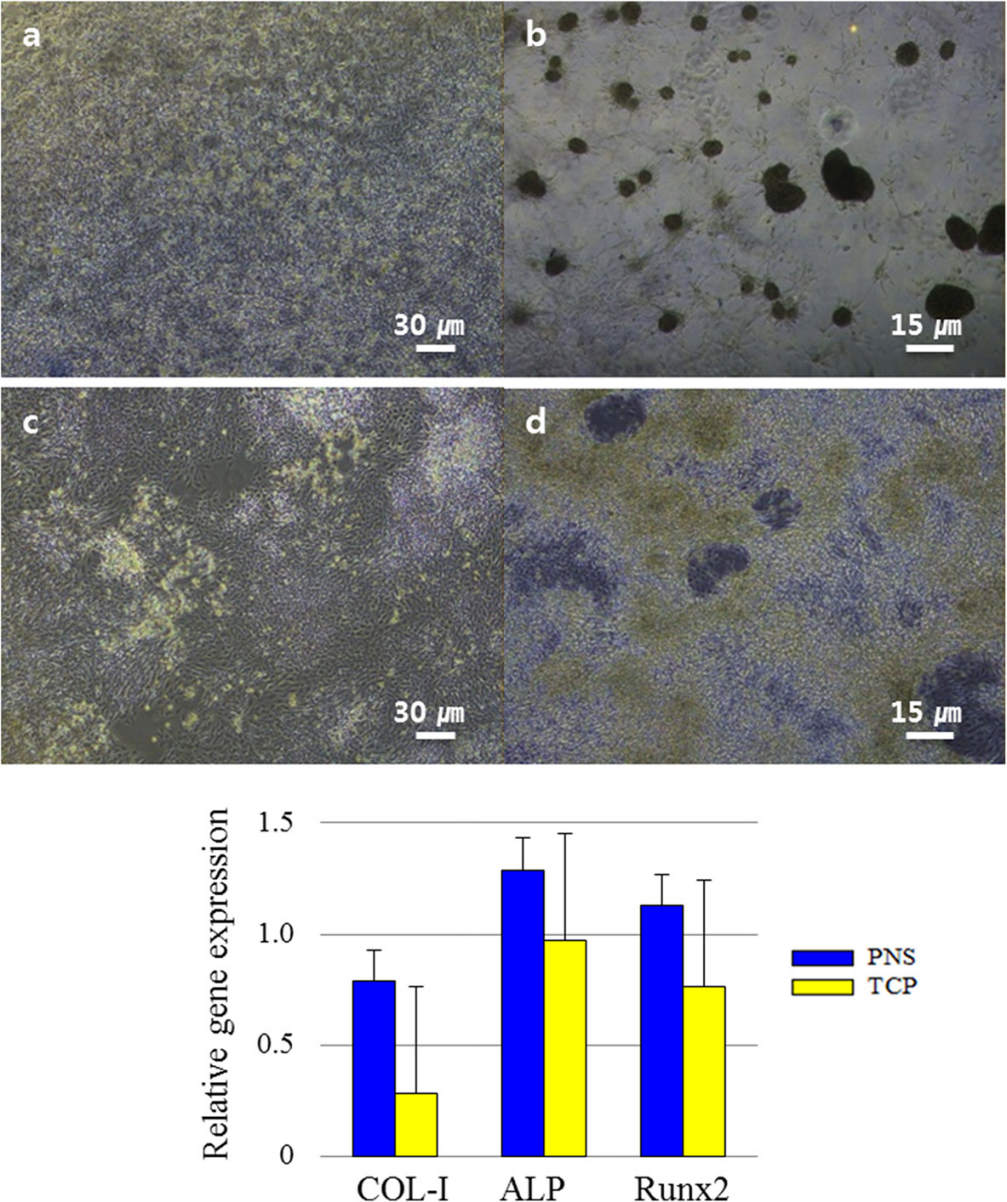
**Micrographs of osteoblast differentiation. (a and b)** Osteoblasts on the polyaniline nanoprotrusion structure surface (PNS). **(a)** Before differentiation. **(b)** Seven days after addition of differentiation medium. **(c and d)** Osteoblasts on the tissue culture plate (TCP). **(c)** Before differentiation. **(d)** Seven days after addition of differentiation medium. The scale bars in **(a)** and **(c)** are 30 μm, and those in **(b)** and **(d)** are 15 μm. The bottom graphs show the relative expression levels of the COL-1, ALP and Runx2 osteoblast markers.

The nanostructure affects not only adhesion and proliferation but also differentiation (Figure 6). Figure 6b shows that differentiation on the PNS exhibited unique characteristics. Osteoblast mineralisation was greater than that on TCP. Calcium deposits, round and sporadic clusters, appeared frequently and appreciable in the images. Osteogenesis-related genes, particularly COL-I, were expressed at higher levels on the PNS than the TCP. These results were consistent with the cell adhesion morphology on the PNS (Figure 4). When the actin filaments are stretched, the nucleus is stimulated and the chromosomes become condensed, which affects DNA synthesis [15]. This stretched actin filament morphology was evident on the PNS as an array of nanostructures 40 nm in diameter.

## Conclusions

We evaluated cell behaviour on a polyaniline nanoprotrusion structure surface. This surface was more hydrophilic than TCP, which enhanced cell adhesion and proliferation. The PNS also increased osteoblast differentiation because the nanoprotrusions affected cell growth and morphology. This surface was fabricated by self-assembly dilute polymerisation, which is a simple and inexpensive method. It showed no cytotoxicity under the conditions tested and was stable under cell culture conditions. The nanoprotrusion structures on the surface affect the shape of the cells and enhance their behaviour.

This study was performed to quantify the effect of polyaniline nanoprotrusion on cell behaviour. Polyaniline is a very effective conducting polymer. The results of the present study will be useful in electrical signal to cell [16]. Our findings suggest that polyaniline surfaces with nanoprotrusions can be applied to culture plates to influence the behaviour of cells seeded thereon.

## Competing interests

The authors declare that they have no competing interests.

## Authors’ contributions

HN designed and performed the experiments and data analysis. TA designed the experiments, contributed to discussion of the data analysis and organised the final manuscript. GL monitored and organised the final manuscript. All authors read and approved the final manuscript.
